# Supplementary material for: Rationally Designed Pyrimidine Compounds: Promising Novel Antibiotics for the Treatment of Staphylococcus aureus-Associated Bovine Mastitis
Source: Antibiotics (Basel). 2023 Aug 21;12(8):1344. doi: 10.3390/antibiotics12081344 (PMC10451377; doi:10.3390/antibiotics12081344)
Supplement: Supplementary file 1 [file antibiotics-12-01344-s001.zip › antibiotics-2562027-supplementary.pdf]

SUPPLEMENTARY INFORMATIONS

Rationally Designed Pyrimidine Compounds: Promising Novel Antibiotics for the Treatment of *Staphylococcus aureus*-Associated Bovine Mastitis.

Guillaume Millette[1#], Evelyne Lacasse[1#], Renaud Binette[2#], Véronique Belley[1], Louis-Philippe Chaumont[1], Céline Ster[1,5], Francis Beaudry[3], Kumaraswamy Boyapelly[4], Pierre-Luc Boudreault[4*], François Malouin[1]**

[1] Département de biologie, Faculté des sciences, Université de Sherbrooke, Sherbrooke, QC, Canada.
[2] Département de chimie, Faculté des sciences, Université de Sherbrooke, Sherbrooke, QC, Canada.
[3] Département de biomédecine vétérinaire, Faculté de médecine vétérinaire, Université de Montréal, St-Hyacinthe, QC, Canada.
[4] Département de pharmacologie et physiologie, Faculté de médecine et sciences de la santé, Université de Sherbrooke, Sherbrooke, QC, Canada.
[5] Present address: Sherbrooke Research and Development Centre, Agriculture and Agri-Food Canada, Sherbrooke, QC J1M 0C8, Canada, Sherbrooke, QC, Canada.

[#] These authors have contributed equally.

* Chemistry correspondence: pierre-luc.boudreault@usherbrooke.ca
** Biology correspondence: francois.malouin@usherbrooke.ca

List of contents

Figure S1. PC371 in gene reporter assay. 3
Figure S2. Measure of the CC_{50} using cells viability assays. 4
Figure S3. Hepatic and plasmatic stability of PC206. 5
Figure S4. Safety assessment of PC206 in cows. 6
Figure S5. UPLC-MS chromatogram of PC206. 7
Figure S6. ^{1}H NMR spectra of PC206. 8
Figure S7. ^{13}C NMR spectra of PC206. 9
Figure S8. UPLC-MS chromatogram of PC371. 10
Figure S9. ^{1}H NMR spectra of PC371. 11
Figure S10. ^{13}C NMR spectra of PC371. 12
Figure S11. UPLC-MS chromatogram of PC372. 13
Figure S12. ^{1}H NMR spectra of PC372. 14
Figure S13. ^{13}C NMR spectra of PC372. 15
Figure S14. UPLC-MS chromatogram of PC383. 16
Figure S15. ^{1}H NMR spectra of PC383. 17

Figure S16. ^{13}C NMR spectra of PC383. 18

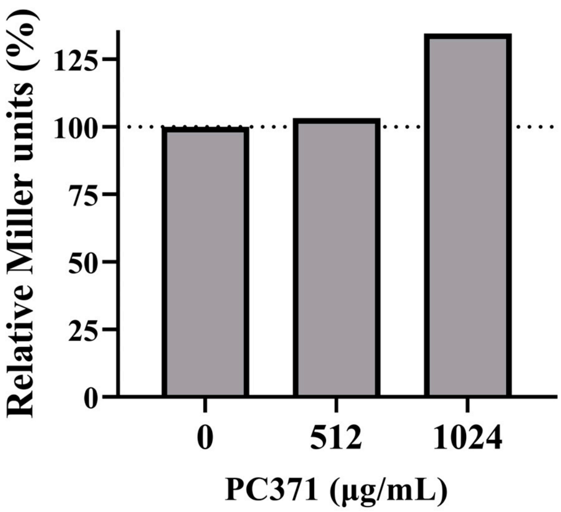

Figure S1. PC371 in gene reporter assay. PC371 at high doses (512 and 1024µg/mL) did not decrease the Miller units. Data was collected from one experiment.

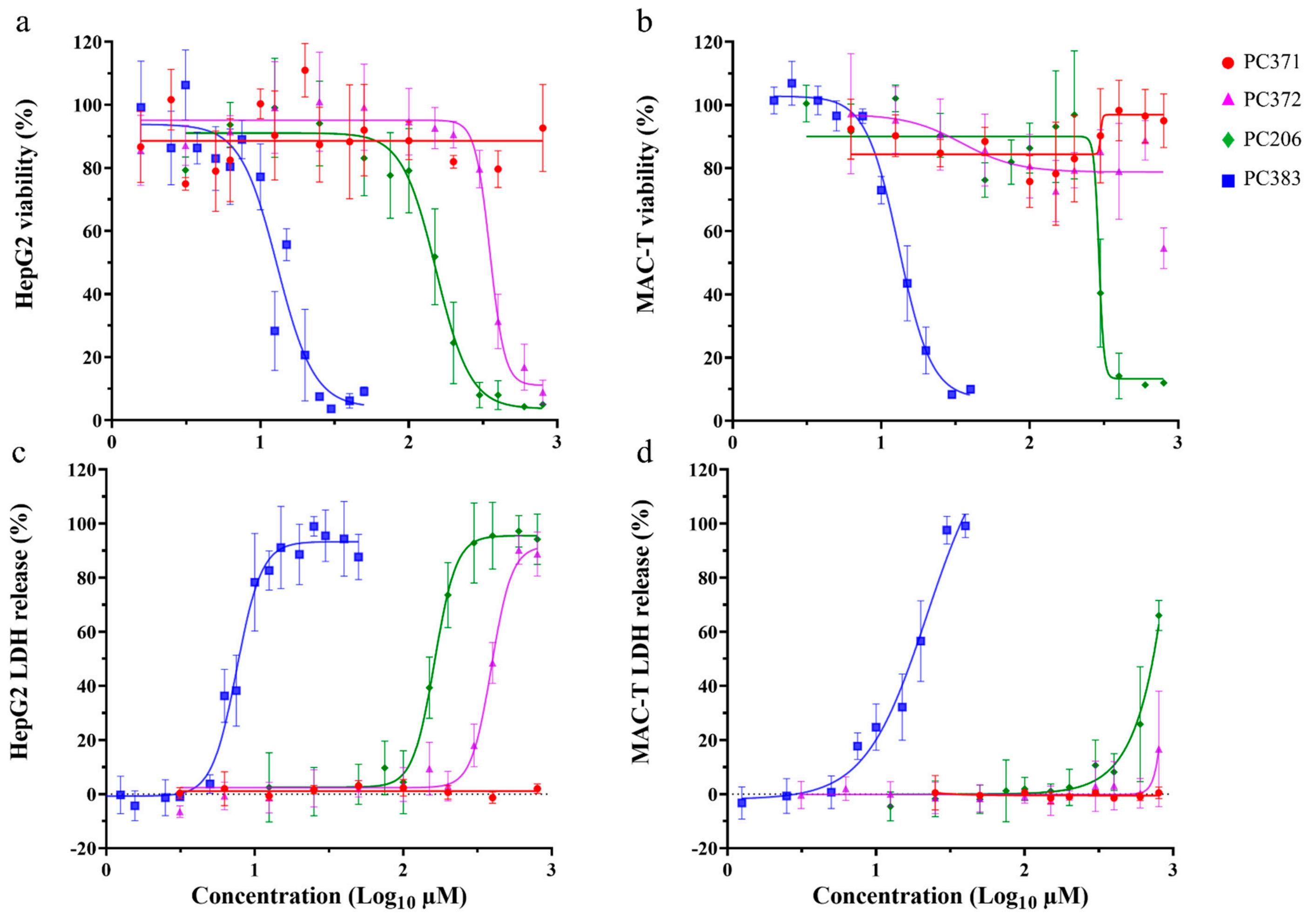

Figure S2. Measure of the CC_{50} using cells viability assays. MTT assay was done with HepG2 (a) and MAC-T (b) cells by increasing the concentration of pyrimidine compounds (PC371, PC372, PC206 and PC383). IC_{50} of at least two assays and three replicates' values were measured with Graph Pad prism 9.4.1. The same has been made with the LDH release assay with HepG2 cells (c), MAC-T cells (d).

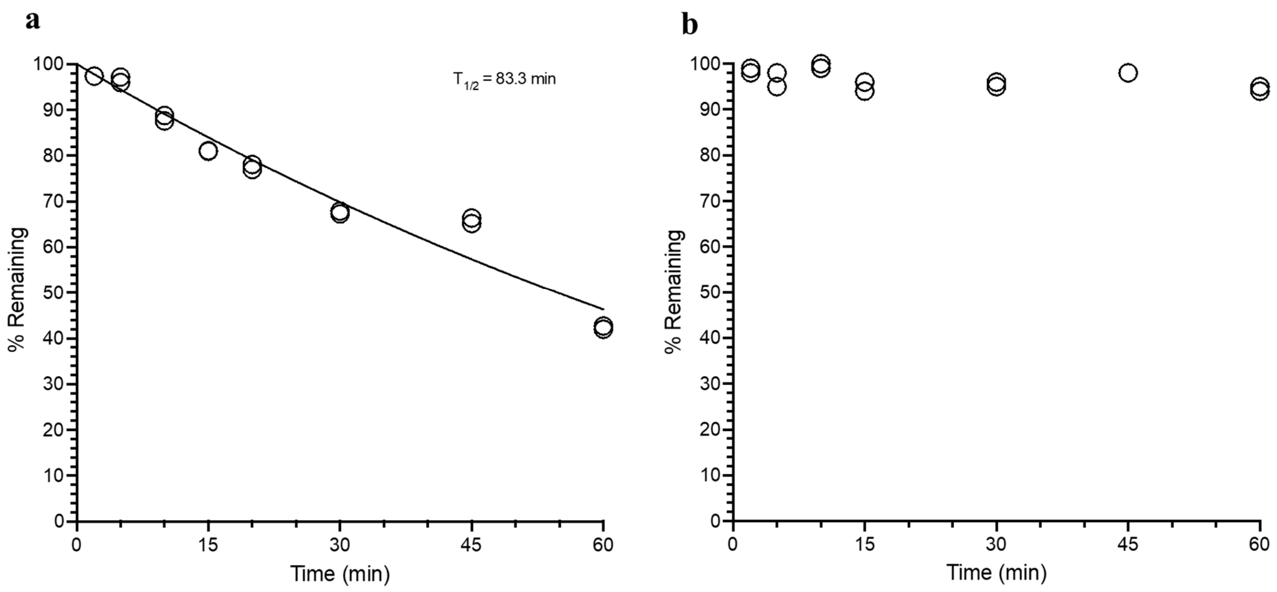

Figure S3. Metabolic and plasmatic stability of PC206. Metabolic stability of PC206 was evaluated over a time period of 60 minutes. For the metabolic stability (a), 0.5 mg/mL of S9 CD-1 female mice liver fraction proteins were exposed to 10 µM of PC206. PC206's half-life was determined to be 83.3 minutes. Regarding the plasmatic stability (b), 10 µM of PC206 was exposed to CD-1 female mice plasma. For timepoints evaluated, PC206 is stable in plasma.

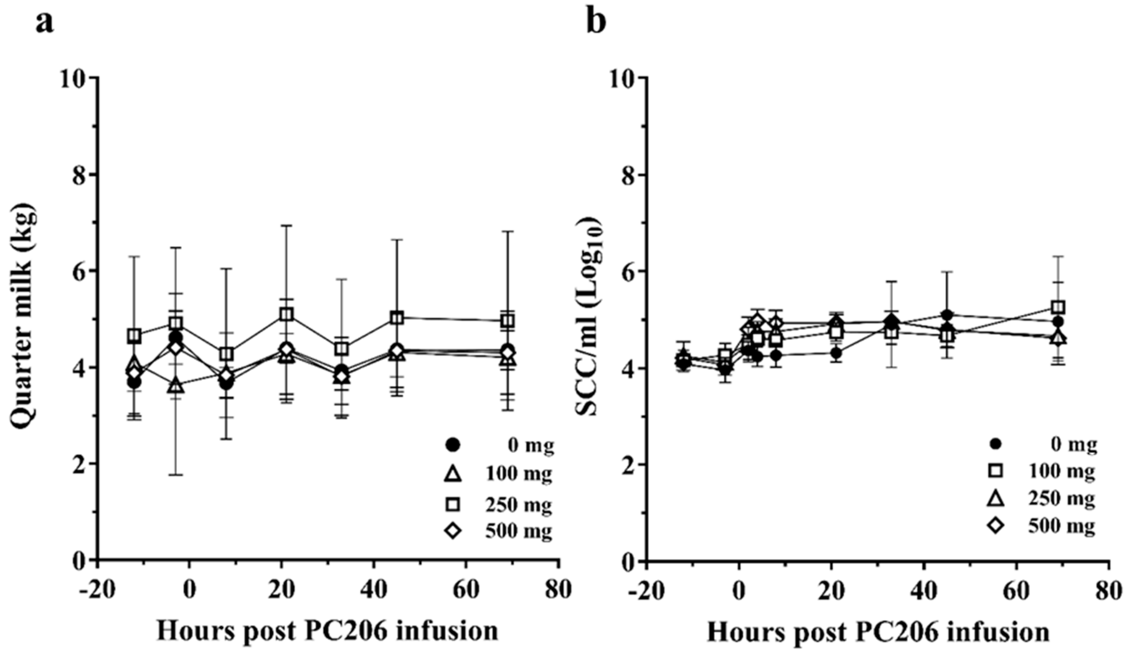

Figure S4. Safety assessment of PC206 in cows. Milk production (a) and somatic cell counts (SCC) (b) were measured as surrogate markers for the onset of inflammation 12 hours before and during 69 hours after PC206 IM infusion given immediately after the morning milking. Body temperature, SCC, quarter milk production and milk appearance did not differ significantly when comparing the three doses to the untreated control group (0 mg).

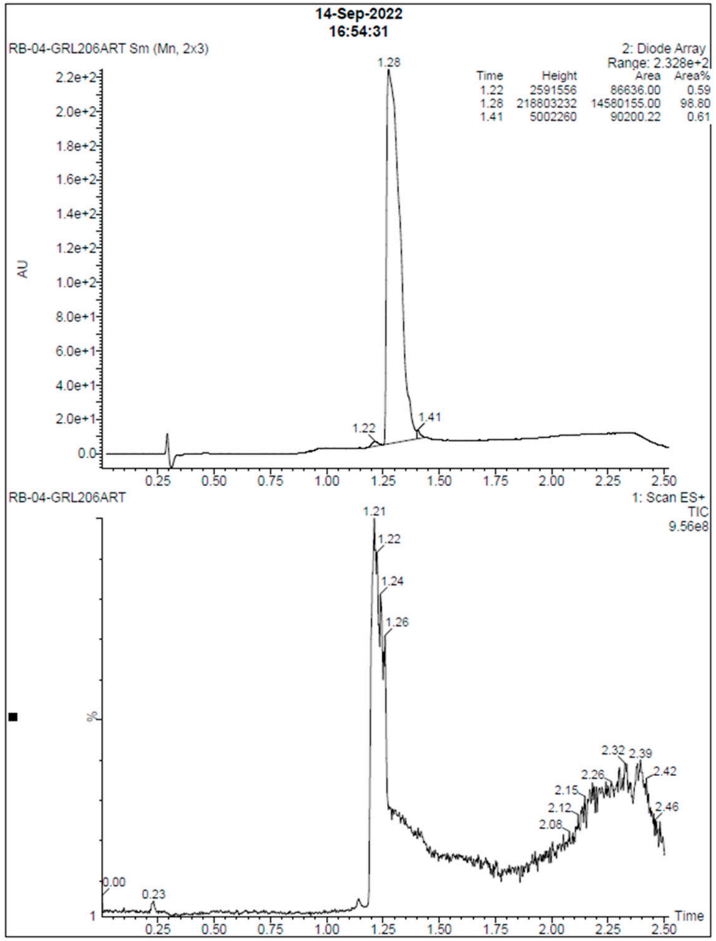

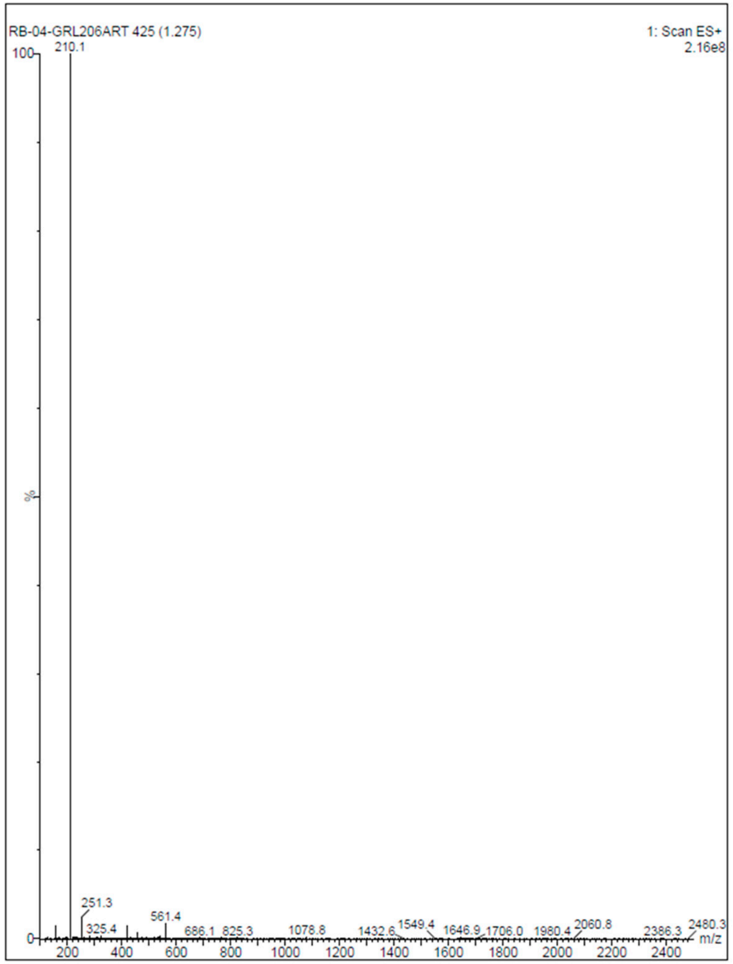

Figure S5. UPLC-MS chromatogram of PC206.

^{1}H NMR (400 MHz, DMSO-d_6) δ ppm 0.84 (t, J=6.50 Hz, 3 H) 1.17 - 1.33 (m, 6 H) 1.45 (quin, J=7.00 Hz, 2 H) 3.12 (q, J=5.50 Hz, 2 H) 4.98 (s, 1 H) 6.77 (br. s., 2 H) 6.94 (br. s., 2 H) 7.31 (br. s., 1 H)

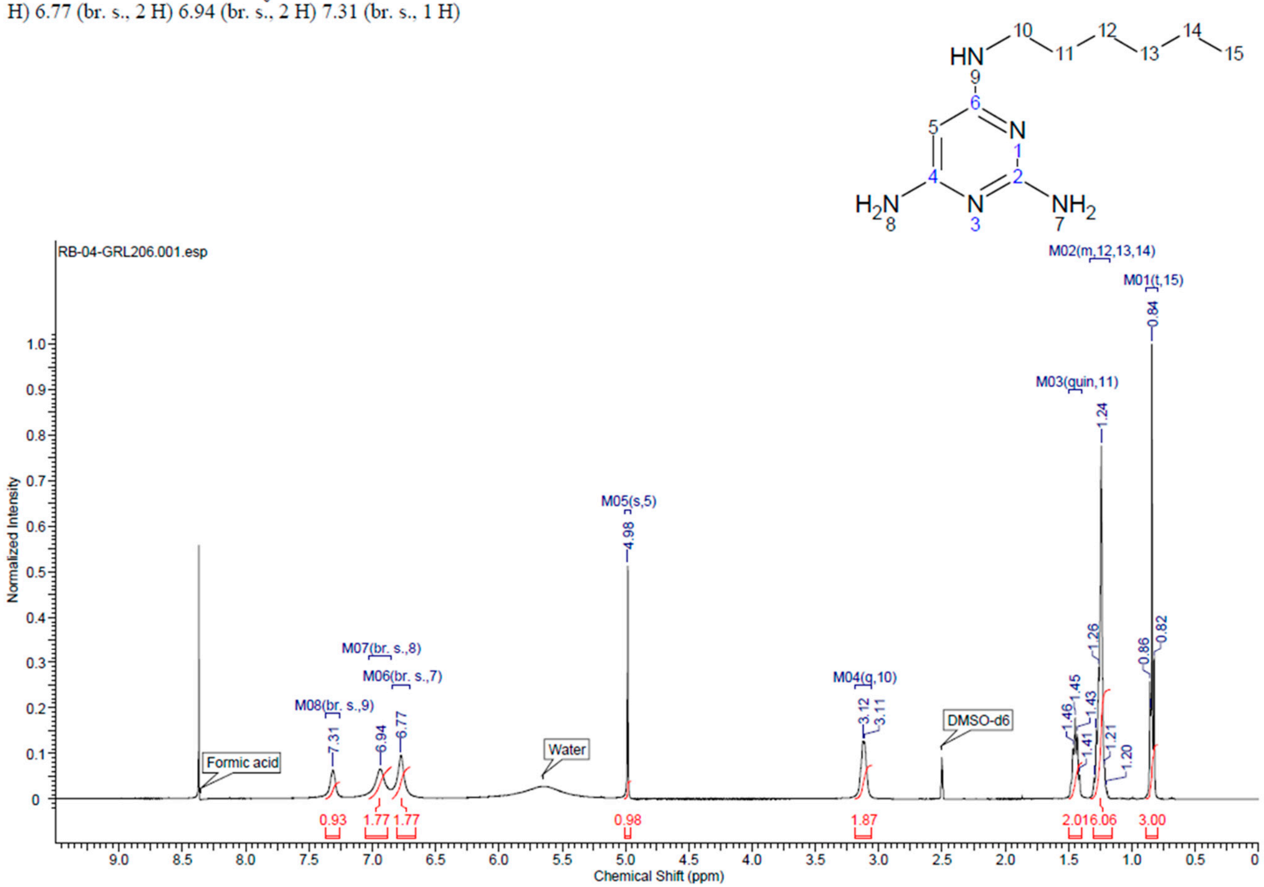

Figure S6. ^{1}H NMR spectra of PC206.

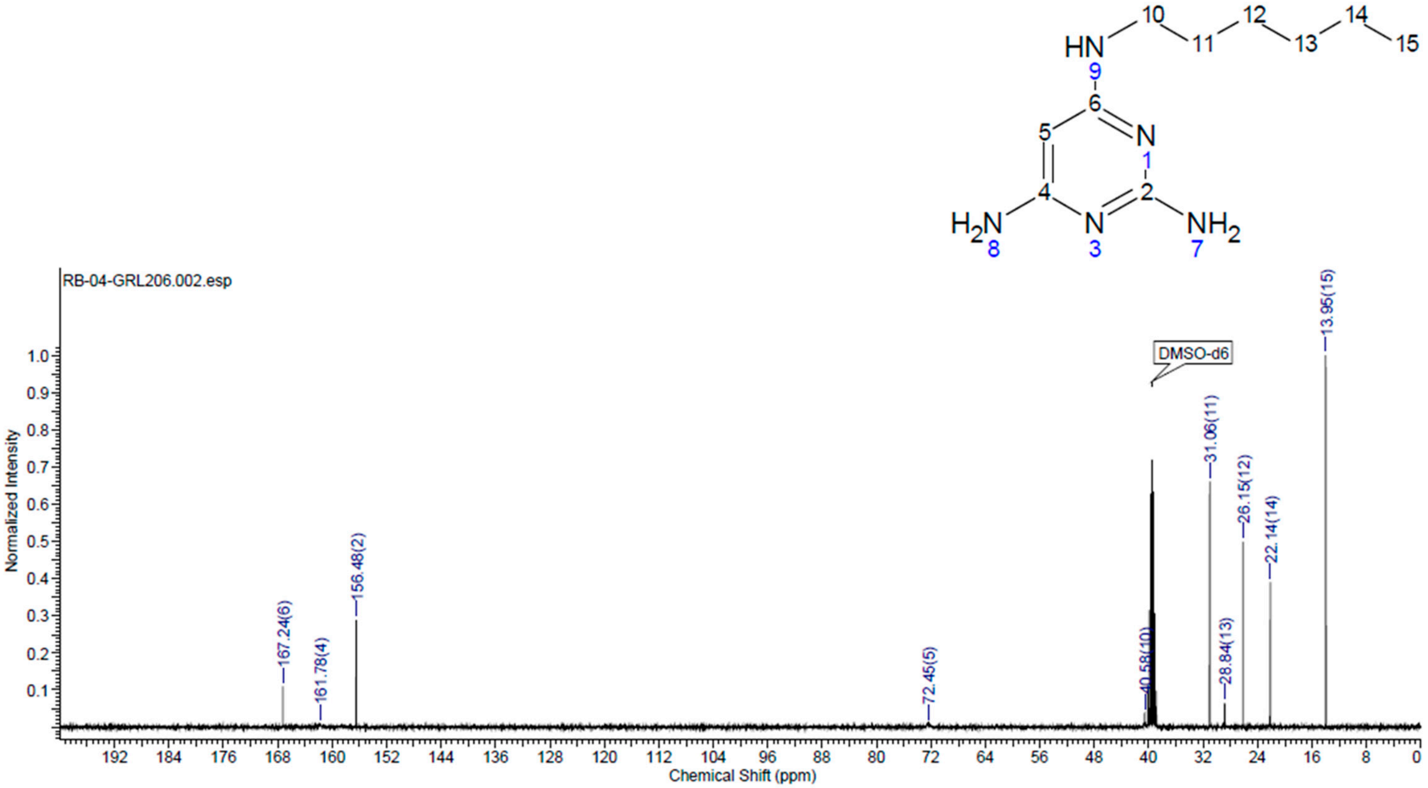

No.	(ppm)	Height	No.	(ppm)	Height	No.	(ppm)	Height	No.	(ppm)	Height	No.	(ppm)	Height
1	167.24	0.1097	3	156.48	0.2884	5	40.58	0.0380	7	28.84	0.0651	9	22.14	0.3907
2	161.78	0.0090	4	72.45	0.0103	6	31.06	0.6603	8	26.15	0.4987	10	13.95	1.0000

Figure S7. ^{13}C NMR spectra of PC206.

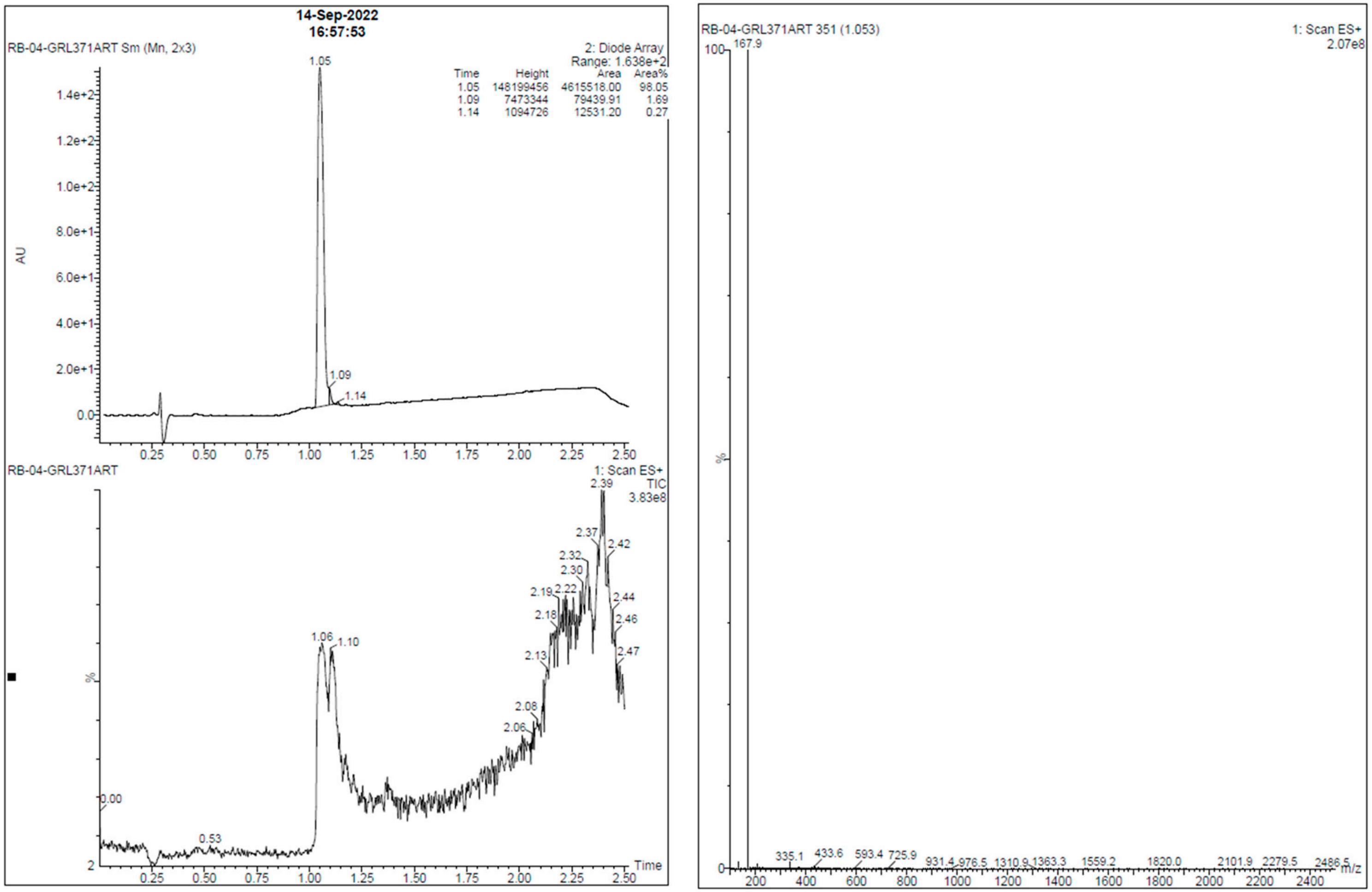

Figure S8. UPLC-MS chromatogram of PC371.

^{1}H NMR (400 MHz, DMSO-d_6) δ ppm 0.86 (t, J=7.43 Hz, 3 H) 1.47 (sxt, J=7.28 Hz, 2 H) 3.07 (q, J=6.43 Hz, 2 H) 4.90 (s, 1 H) 6.18 (br. s., 4 H) 6.72 (br. s., 1 H)

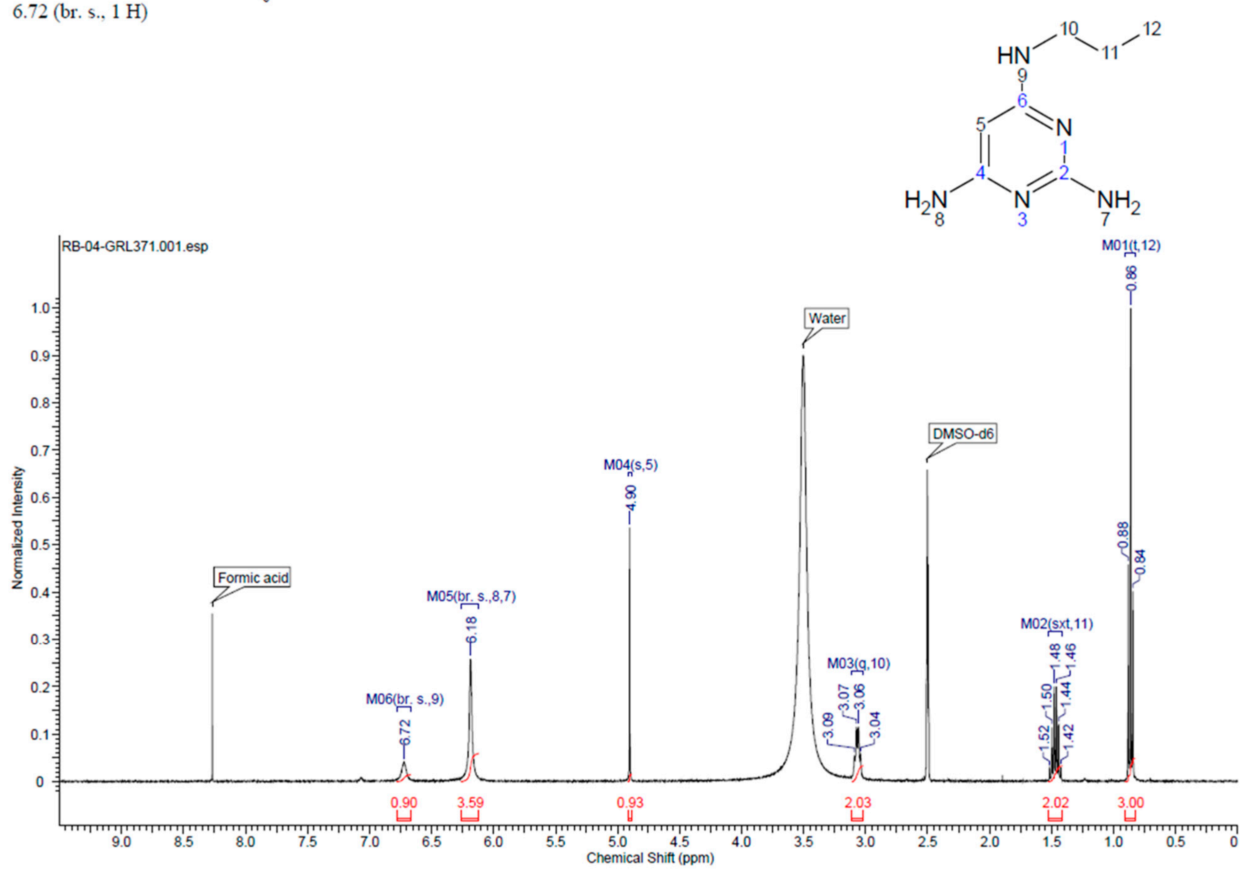

Figure S9. ^{1}H NMR spectra of PC371.

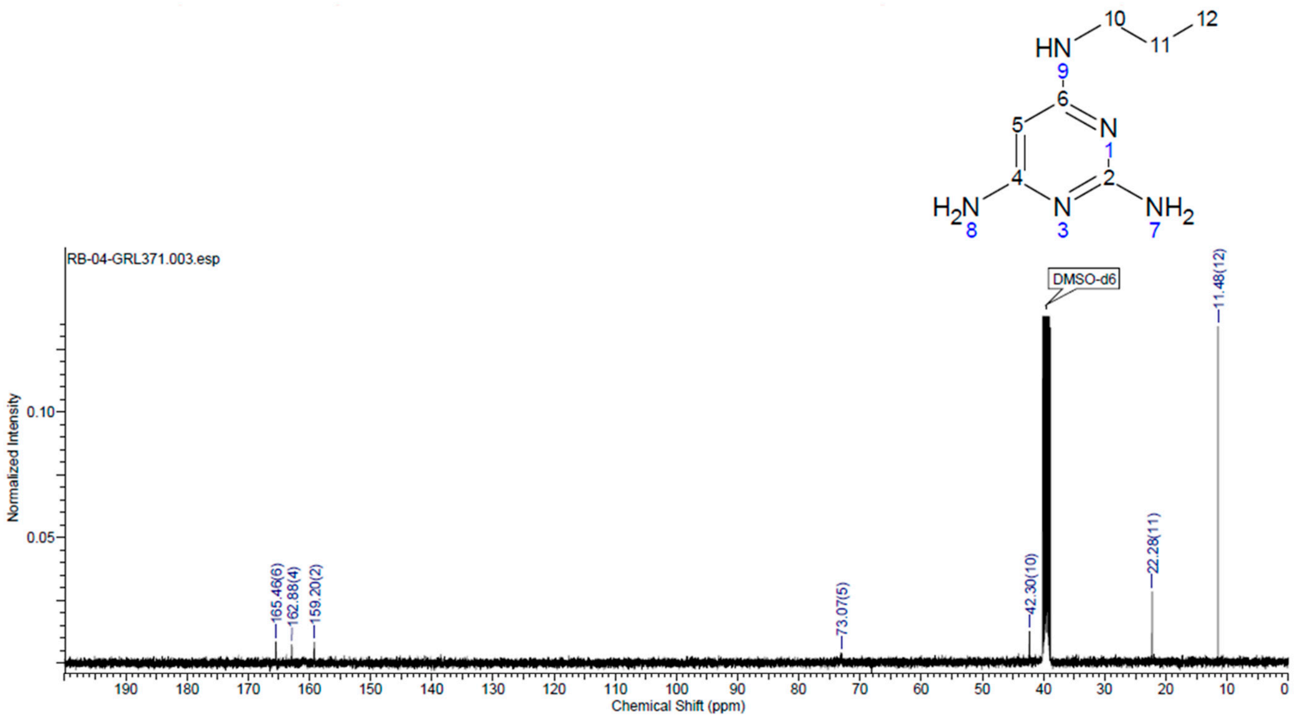

No.	(ppm)	Height
1	165.46	0.0086
2	162.88	0.0074
3	159.20	0.0084
4	73.07	0.0041
5	42.30	0.0126
6	22.28	0.0285
7	11.48	0.1341

Figure S10. ^{13}C NMR spectra of PC371.

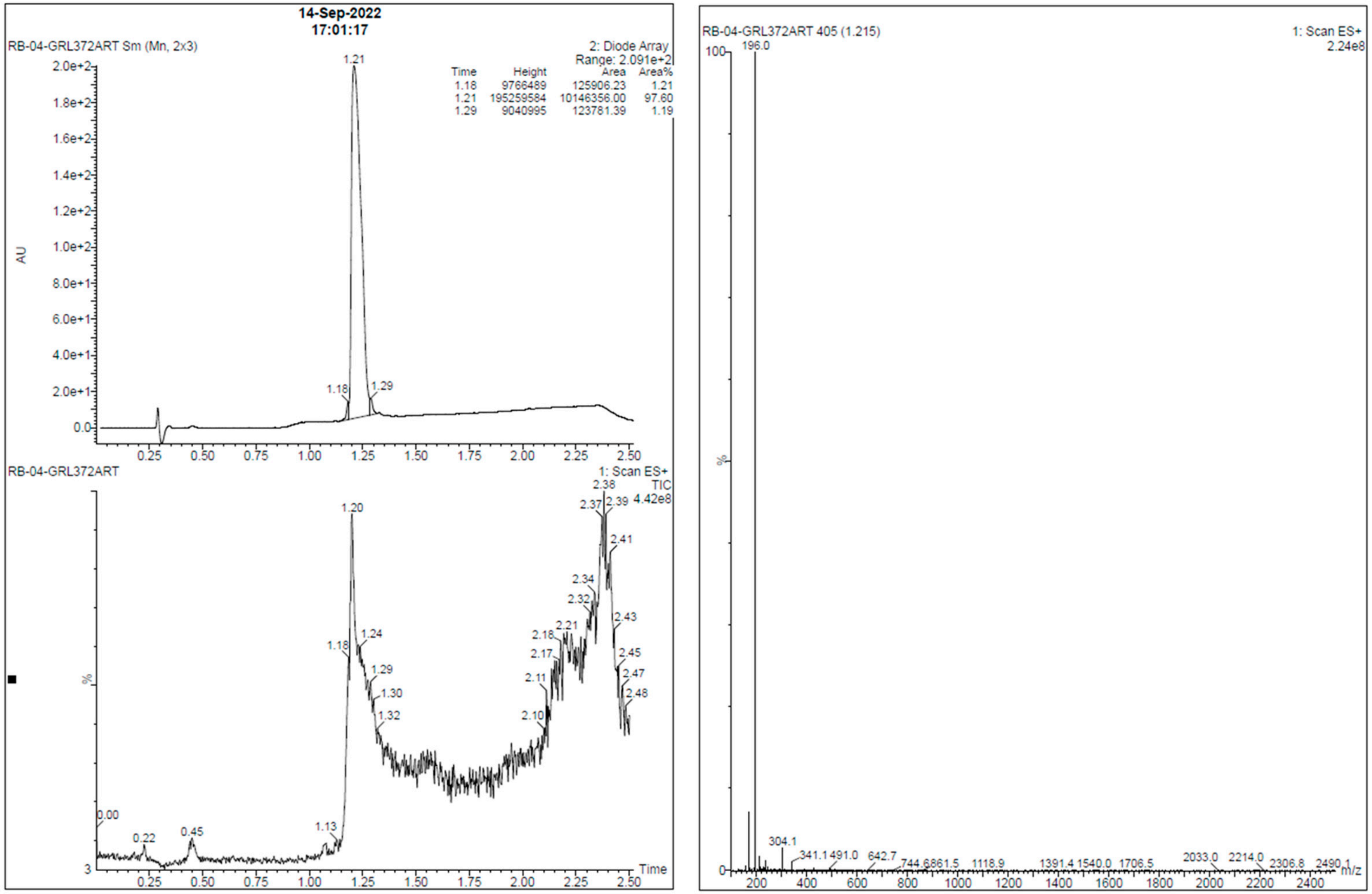

Figure S11. UPLC-MS chromatogram of PC372.

^{1}H NMR (400 MHz, DMSO-d_6) δ ppm 0.87 (t, J=7.00 Hz, 3 H) 1.21 - 1.33 (m, 4 H) 1.46 (quin, J=7.09 Hz, 2 H) 3.12 (q, J=6.00 Hz, 2 H) 4.95 (s, 1 H) 6.57 (br. s., 2 H) 6.66 (br. s., 2 H) 7.11 (br. s., 1 H)

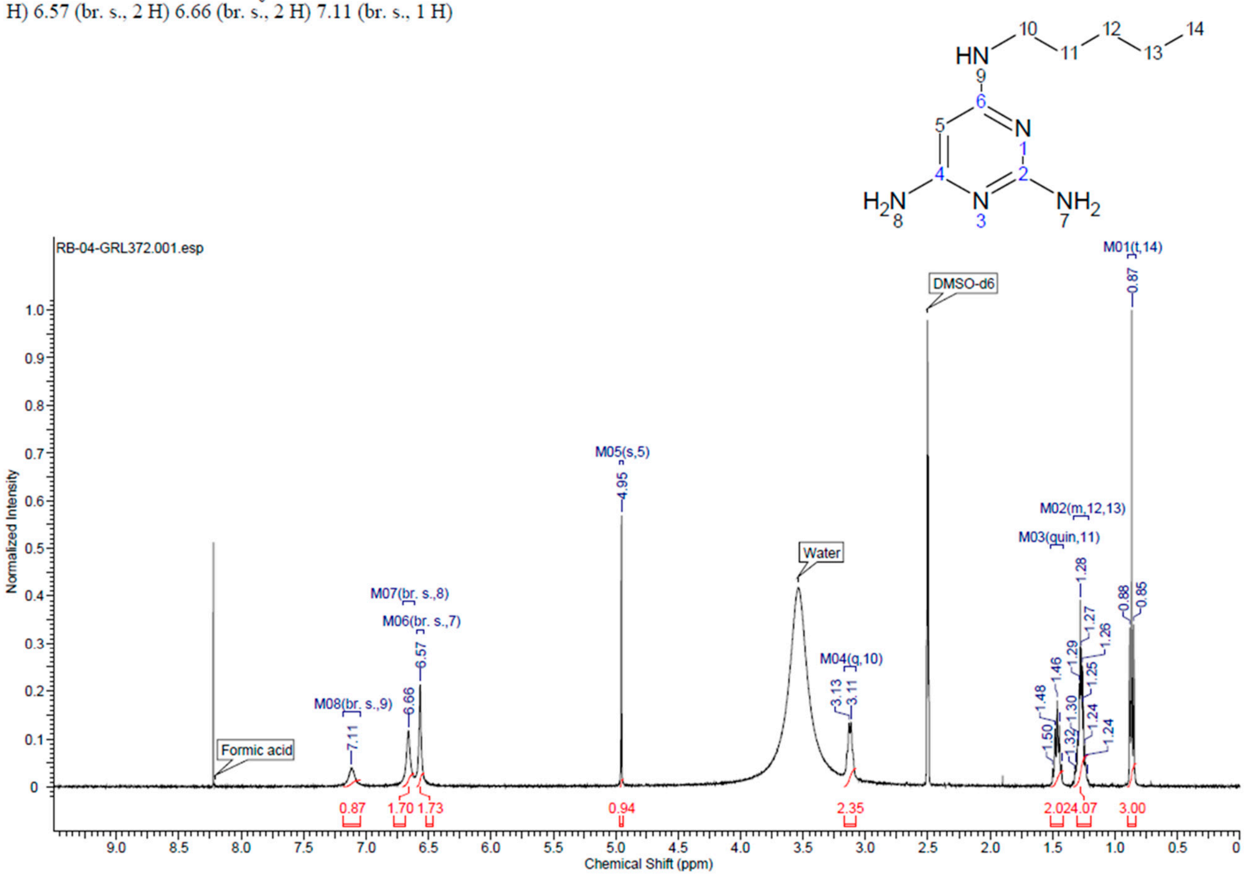

Figure S12. ^{1}H NMR spectra of PC372.

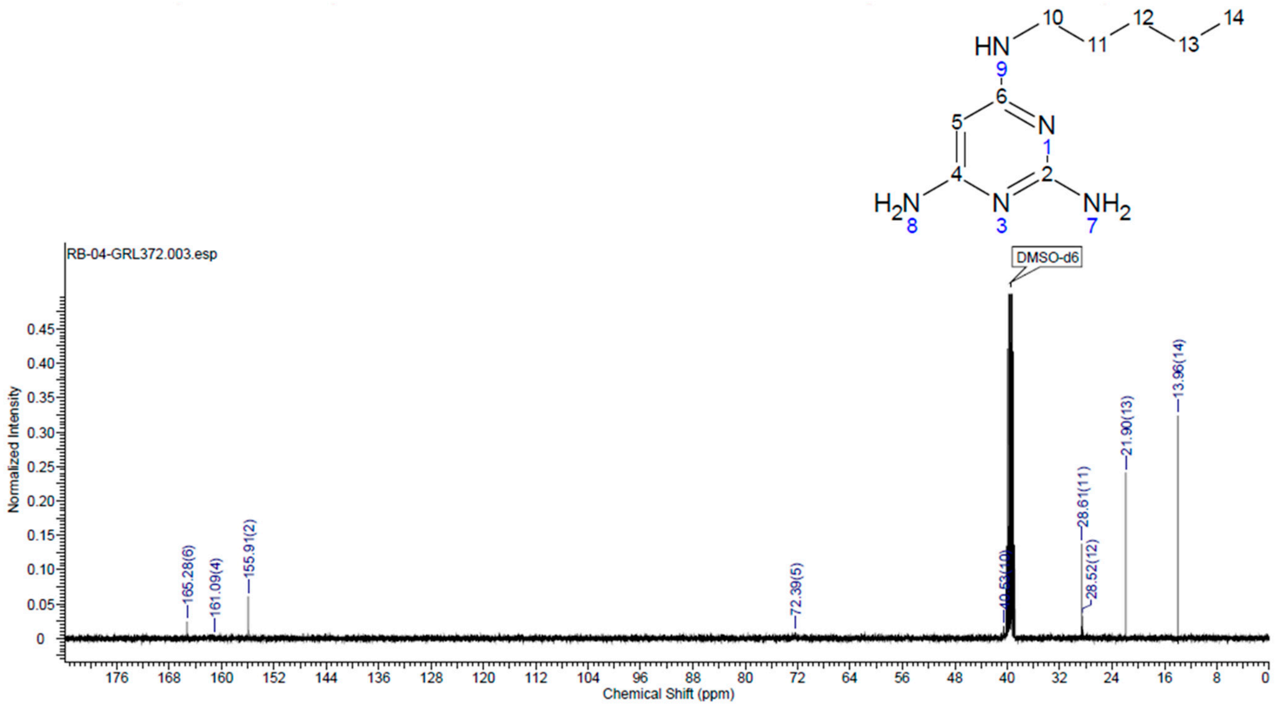

No.	(ppm)	Height
1	165.28	0.0240
2	161.09	0.0055
3	155.91	0.0611
4	72.39	0.0090
5	40.53	0.0174
6	28.61	0.1377
7	28.52	0.0323
8	21.90	0.2410
9	13.96	0.3235

Figure S13. ^{13}C NMR spectra of PC372.

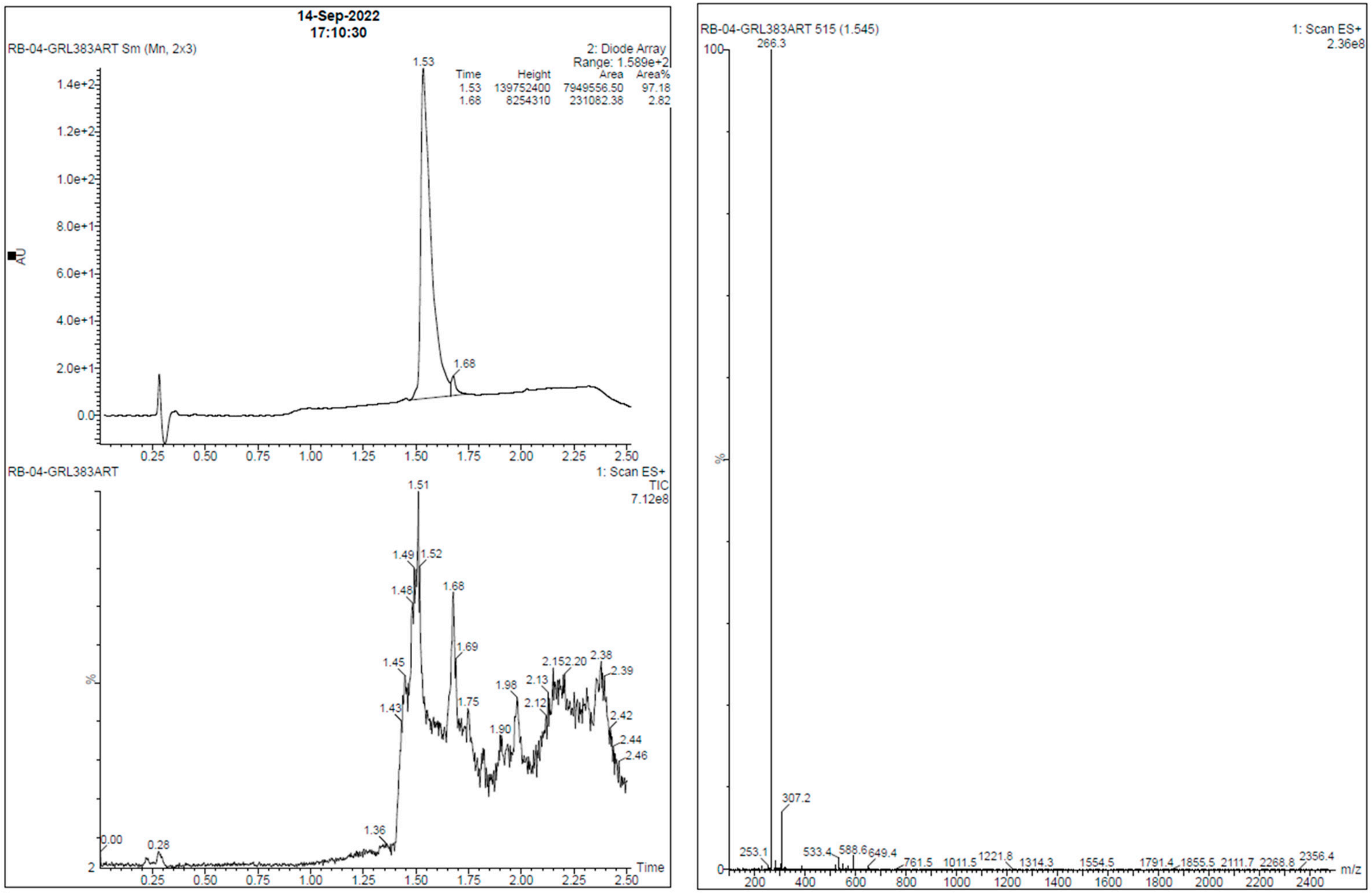

Figure S14. UPLC-MS chromatogram of PC383.

^{1}H NMR (400 MHz, DMSO-d_6) δ ppm 0.85 (t, J=7.00 Hz, 3 H) 1.18 - 1.32 (m, 14 H) 1.46 (quin, J=6.50 Hz, 2 H) 3.14 (q, J=5.00 Hz, 2 H) 4.99 (s, 1 H) 6.82 (br. s., 2 H) 6.98 (br. s., 2 H) 7.38 (br. s., 1 H)

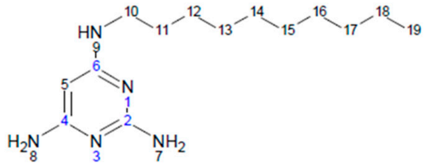

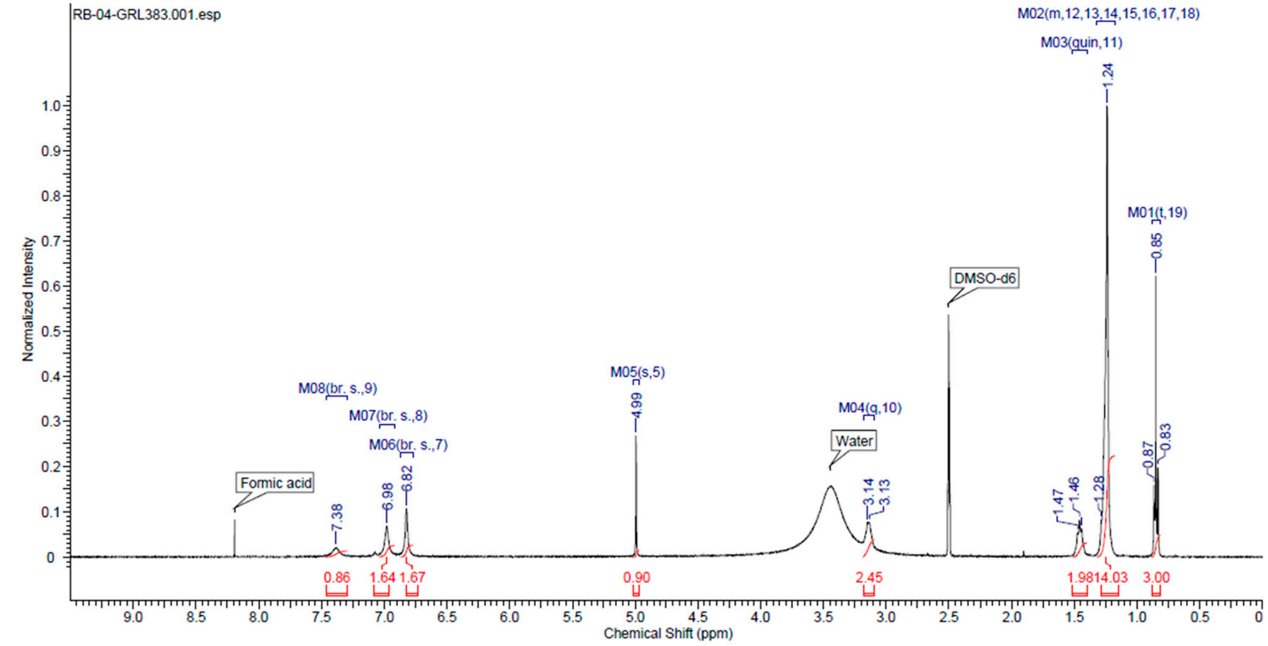

Figure S15. ^{1}H NMR spectra of PC383.

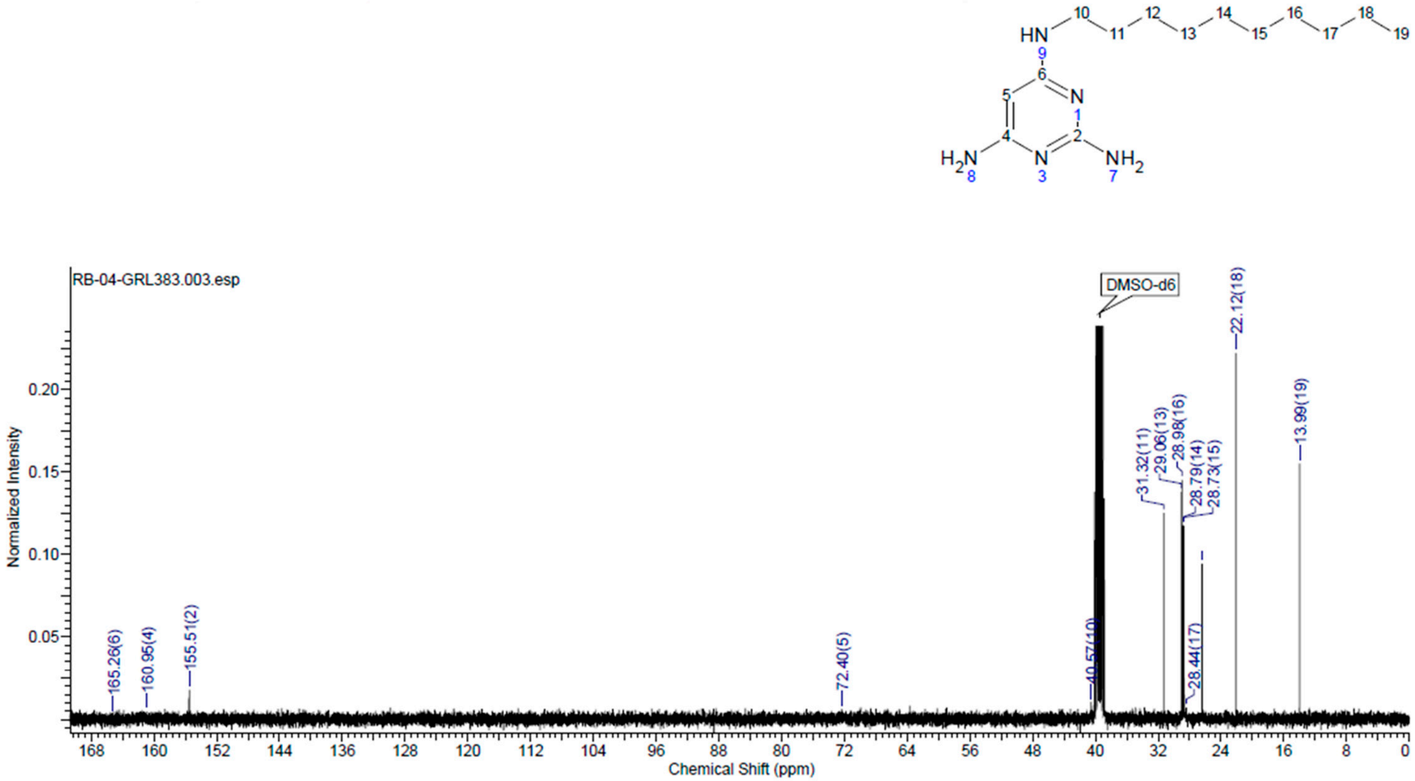

No.	(ppm)	Height	No.	(ppm)	Height	No.	(ppm)	Height	No.	(ppm)	Height	No.	(ppm)	Height
1	165.26	0.0028	4	72.40	0.0060	7	29.06	0.1378	10	28.73	0.1175	13	22.12	0.2223
2	160.95	0.0048	5	40.57	0.0102	8	28.98	0.1454	11	28.44	0.0069	14	13.99	0.1551
3	155.51	0.0177	6	31.32	0.1250	9	28.79	0.1176	12	26.42	0.0945			

Figure S16. ^{13}C NMR spectra of PC383.
